# Latino Parents’ Reactions to and Engagement With a Facebook Group–Based COVID-19 Vaccine Promotion Intervention: Mixed Methods Pilot Study

**DOI:** 10.2196/51331

**Published:** 2024-03-14

**Authors:** Anna I González-Salinas, Elizabeth L Andrade, Lorien C Abroms, Kaitlyn Gómez, Carla Favetto, Valeria M Gómez, Karen K Collins

**Affiliations:** 1 George Washington University Washington, DC United States; 2 California State University Fullerton, CA United States

**Keywords:** COVID-19, misinformation, social media, Latino parents, Spanish, vaccines, digital intervention

## Abstract

**Background:**

Misinformation in Spanish on social media platforms has contributed to COVID-19 vaccine hesitancy among Latino parents. Brigada Digital de Salud was established to disseminate credible, science-based information about COVID-19 in Spanish on social media.

**Objective:**

This study aims to assess participants’ reactions to and engagement with Brigada Digital content that sought to increase COVID-19 vaccine uptake among US Latino parents and their children.

**Methods:**

We conducted a 5-week intervention in a private, moderator-led Facebook (Meta Platforms, Inc) group with Spanish-speaking Latino parents of children aged ≤18 years (N=55). The intervention participants received 3 to 4 daily Brigada Digital posts and were encouraged to discuss the covered topics through comments and polls. To assess participants’ exposure, reactions, and engagement, we used participants’ responses to a web-based survey administered at 2 time points (baseline and after 5 weeks) and Facebook analytics to calculate the average number of participant views, reactions, and comments. Descriptive statistics were assessed for quantitative survey items, qualitative responses were thematically analyzed, and quotes were selected to illustrate the themes.

**Results:**

Overall, 101 posts were published. Most participants reported visiting the group 1 to 3 times (22/55, 40%) or 4 to 6 (18/55, 33%) times per week and viewing 1 to 2 (23/55, 42%) or 3 to 4 (16/55, 29%) posts per day. Facebook analytics validated this exposure, with 36 views per participant on average. The participants reacted positively to the intervention. Most participants found the content informative and trustworthy (49/55, 89%), easy to understand, and presented in an interesting manner. The participants thought that the moderators were well informed (51/55, 93%) and helpful (50/55, 91%) and praised them for being empathic and responsive. The participants viewed the group environment as welcoming and group members as friendly (45/55, 82%) and supportive (19/55, 35%). The 3 most useful topics for participants were the safety and efficacy of adult COVID-19 vaccines (29/55, 53%), understanding child risk levels (29/55, 53%), and the science behind COVID-19 (24/55, 44%). The preferred formats were educational posts that could be read (38/55, 69%) and videos, including expert (28/55, 51%) and instructional (26/55, 47%) interviews. Regarding engagement, most participants self-reported reacting to posts 1 to 2 (16/55, 29%) or 3 to 4 (15/55, 27%) times per week and commenting on posts 1 to 2 (16/55, 29%) or <1 (20/55, 36%) time per week. This engagement level was validated by analytics, with 10.6 reactions and 3 comments per participant, on average, during the 5 weeks. Participants recommended more opportunities for engagement, such as interacting with the moderators in real time.

**Conclusions:**

With adequate intervention exposure and engagement and overall positive participant reactions, the findings highlight the promise of this digital approach for COVID-19 vaccine–related health promotion.

## Introduction

### Background

US Latino individuals have experienced distressing COVID-19 disparities related to morbidity and mortality. Recent studies have demonstrated this heightened risk in Latino adults, who have a 1.5 times greater risk of infection, 2.3 times greater risk of hospitalization, and 1.8 times greater risk of death [1-5]. These increased risks are likely related, in part, to slower vaccine uptake during the pandemic [6,7]. Although adult COVID-19 vaccination rates have increased, with 57.1% of US Latino adults having completed a primary vaccine series as of March 2023, only 8.5% of Latino adults have received a bivalent booster dose, the lowest coverage across racial and ethnic subgroups [8].

What is also alarming is the underrepresentation of Latino children in some age groups among the population vaccinated against COVID-19. The vaccination rates of Latino children continue to lag, with only 5.1% of Latino children aged 6 months to 4 years having completed a 2-dose vaccine series as of December 2022 and 28.8% of Latino children aged 5 to 11 years, 57.8% of Latino adolescents aged 12 to 15 years, and 70.4% of Latino adolescents aged 16 to 17 years having completed the primary series as of August 2022 [9,10]. Booster dose coverage among Latino children is also worrisome, with only 4.6% of Latino children aged 5 to 11 years, 20.7% of Latino adolescents aged 12 to 15 years, and 32.3% of Latino adolescents aged 16 to 17 years having received a booster dose as of August 2022 [9,10]. These low rates are particularly concerning, given that research has demonstrated an increased risk among Latino children and adolescents aged <18 years for COVID-19 infection, hospitalization, multisystem inflammatory syndrome, and related health complications [11-16]. These findings emphasize the need for targeted interventions to mitigate these disproportionate risks and increase immunization rates.

Research has revealed that COVID-19 vaccine hesitancy has been a significant obstacle to reducing these risks for US Latino communities [4,17-19], especially among Latino parents, who have exhibited considerable reluctance toward vaccinating their children against COVID-19 [20-23]. This hesitancy has been partly fueled by exposure to misinformation regarding COVID-19 vaccines, primarily through social media networks [20,24-27]. This exposure has created more mistrust and concerns among Latino parents regarding the safety and efficacy of COVID-19 vaccines for children [28-32], and studies have shown an association between exposure to misinformation on social media and increased hesitancy and decreased intention to vaccinate [33-39].

Although there have been efforts to address vaccine hesitancy among Latino individuals, including communication campaigns and community-based outreach initiatives [25,40-42], few social media–based intervention studies have focused on Latino parents. Ramirez and colleagues [43] implemented a Facebook (Meta Platforms, Inc)–based pilot intervention to promote COVID-19 vaccine uptake among rural community residents in South Texas, whereby intentions to vaccinate were assessed following exposure to video testimonials that acknowledged misinformation and promoted vaccination using peer modeling. Although not explicitly focused on parents, this study demonstrated significantly higher vaccine intentions following exposure to such video testimonials compared with exposure to standard Centers for Disease Control and Prevention advertisements. In addition, a study by Panameno and colleagues [44] assessed Latino parents’ experiences with *MiVacunaLA*, a mobile phone–delivered digital intervention for improving Latino parents’ vaccination intentions and Latino children’s vaccination rates. The study found that digital technology was beneficial for delivering language-tailored and culturally tailored pediatric COVID-19 vaccine information to Latino parents, which addressed their specific informational needs and increased their confidence in COVID-19 vaccines. This study highlighted the importance of concise, accessible information delivered by trusted sources, such as videos portraying physicians or community health workers, to overcome barriers to health literacy or other barriers to COVID-19 vaccination. Furthermore, this study revealed a persistent need for culturally tailored information about COVID-19 vaccines and boosters, specifically information delivered in Spanish and through trusted sources.

### Objectives

In response to information gaps among US-based Spanish-speaking audiences, we established Brigada Digital de Salud in 2021 to disseminate accurate, credible, and science-based COVID-19 information and prevention messages in Spanish based on the latest research and public health guidelines. Brigada Digital aims to serve as a social media resource for Spanish-speaking individuals who want to make sound decisions about their health and the health of their families in the face of web-based misinformation [45]. This paper details the findings from a 5-week study among Latino parents in a private Facebook group to assess their reactions to and engagement with Brigada Digital social media content and to elicit their recommendations for future iterations of the intervention.

## Methods

### Participant Recruitment

Eligible study participants included Latino adults aged ≥18 years who were parents of at least 1 child aged <18 years, spoke fluent Spanish, and reported using Facebook at least once daily. We recruited participants through 8 targeted Facebook advertisements between August 12 and August 22, 2022. All interested individuals completed an 8-item screener to determine eligibility, and eligible participants were automatically directed to an informed consent form and a baseline survey administered using Qualtrics (Qualtrics International Inc). The participants who completed the baseline survey were assigned to either the intervention Facebook group (N=55) that received Brigada Digital content or the control Facebook group (n=65) that received only a link to the Centers for Disease Control and Prevention web-based COVID-19 information in Spanish (standard of care) but did not receive the intervention content. This paper discusses the reactions and engagement of only the participants in the intervention group.

### Intervention Overview

**Figure 1:**
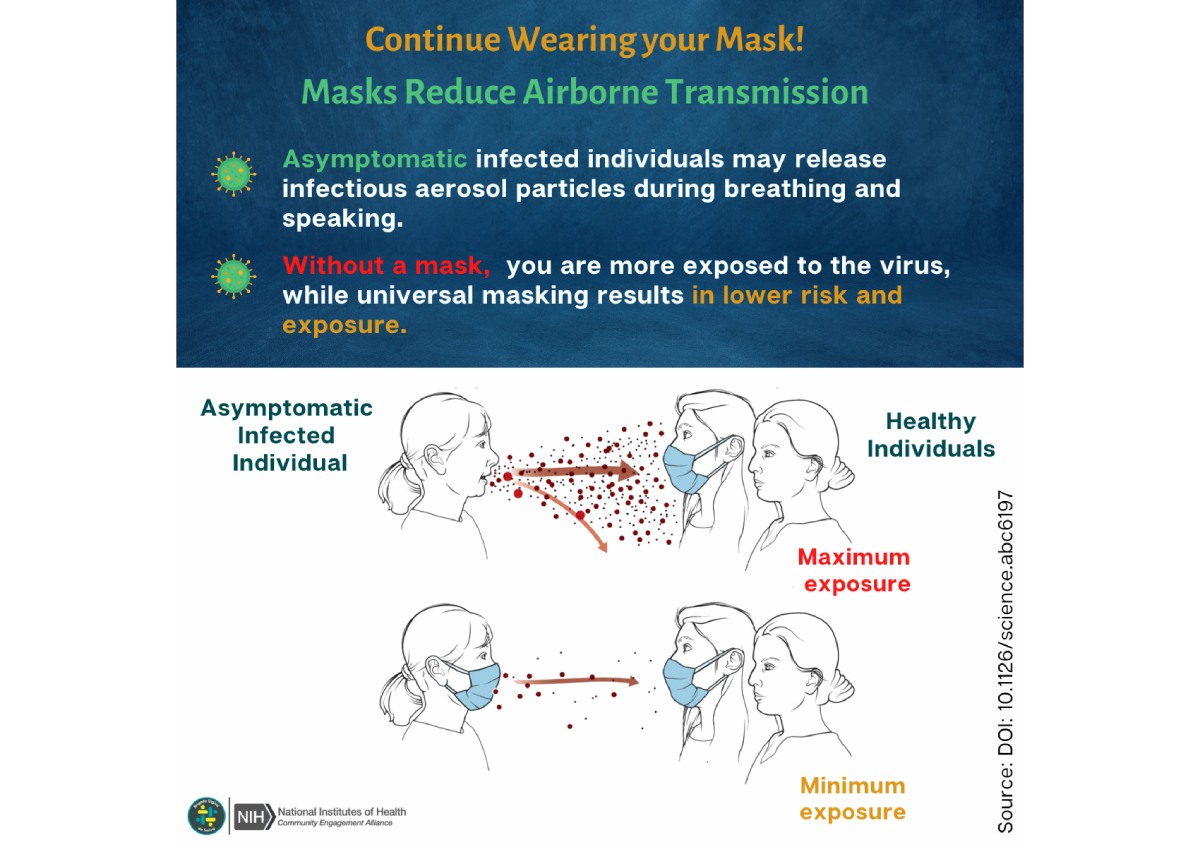
Brigada Digital social media post using Spanish audio narration to emphasize the importance of wearing a mask to reduce respiratory viral transmission.

A 5-week intervention delivered through Facebook groups was created to educate participants on the risks of COVID-19, COVID-19 prevention methods, and the importance of adult and child vaccination. The intervention aimed to address common misinformation often spread through social media. Intervention content development was informed by the Theory of Planned Behavior, according to which beliefs about vaccination, social norms, perceived control over vaccination, and attitudes about vaccination influenced COVID-19 vaccination intentions [46]. The intervention content was developed before the study and was carefully crafted to be accessible to a broad Latino audience, regardless of education or health literacy levels. For example, scientific concepts were explained in simple terms, prevention recommendations were accompanied by visual aids, and the text was audio narrated in Spanish (Figure 1).

Once participants were accepted into the Facebook group, they received prerecorded welcome videos from moderators, 3 posts, and an overview of group expectations and guidelines and were invited to introduce themselves to the group. This point onward, posts were scheduled to be shared with the group on a daily basis. In total, 101 posts were shared during the 5-week period, with at least 2 posts per day being educational and the remainder alternating between posts intended to counter COVID-19 misinformation and posts intended to engage group members.

**Figure 2:**
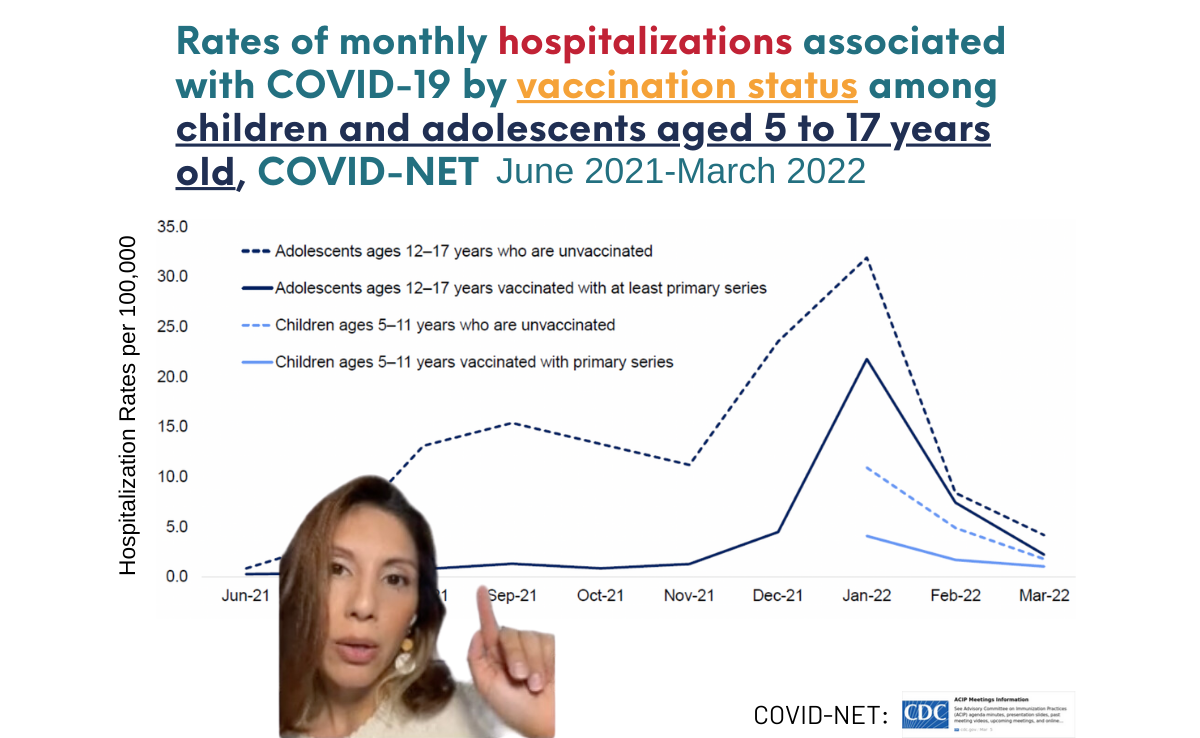
Brigada Digital TikTok video featuring a Latina health expert explaining the cumulative monthly COVID-19-related hospitalizations among children ages 5-17 years old from June 2021 to March 2022.

The content was delivered using various formats, such as a narrated slide carousel, animated images with text and music, and videos featuring health professionals and subject matter experts (Figure 2).

**Figure 3:**
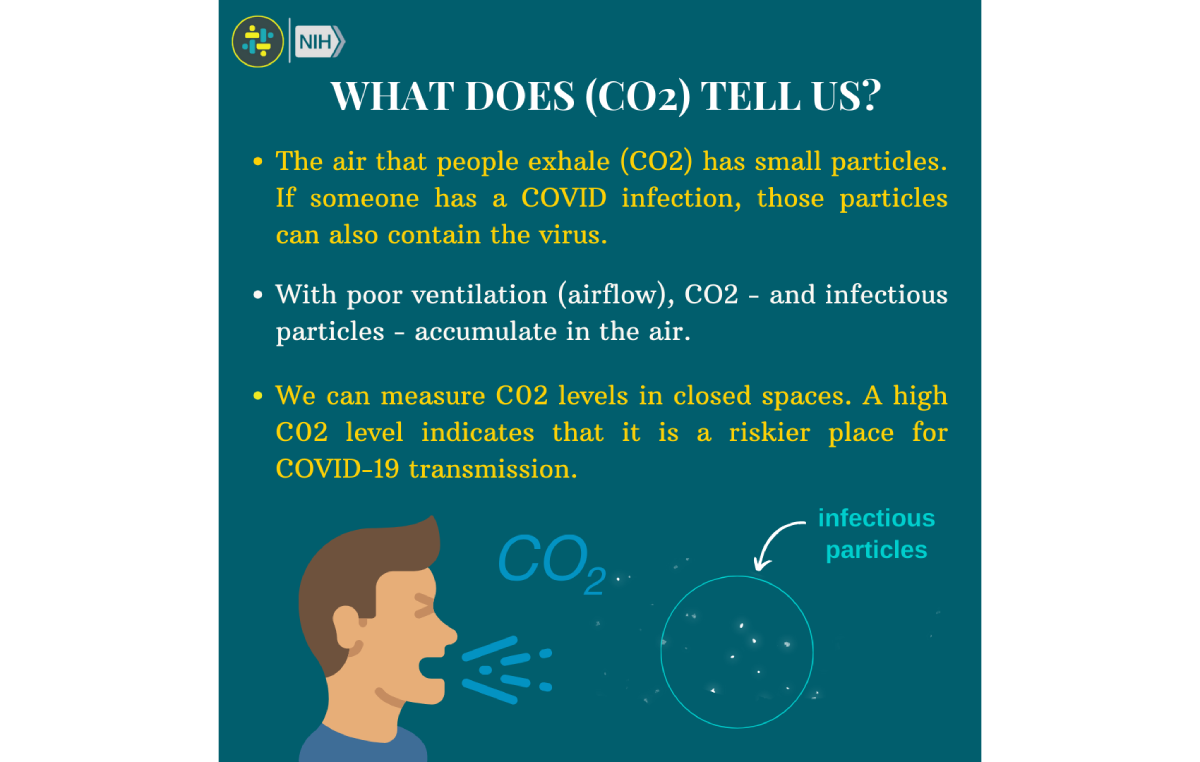
Brigada Digital educational post using animated images with text and Spanish audio narration to educate about the link between carbon dioxide levels and COVID-19 risk and provide guidelines for indoor mask use. NIH: National Institutes of Health.

Furthermore, the post content was ordered to progress through various themes related to COVID-19, beginning with fundamental scientific concepts behind disease transmission and then covering topics with increasing complexity. For example, we incorporated content explaining the connection between carbon dioxide levels in indoor environments and COVID-19 transmission risk, which educated participants regarding the risks associated with gathering indoors in crowded spaces and encouraged them to improve ventilation indoors to reduce their risk (Figure 3).

**Figure 4:**
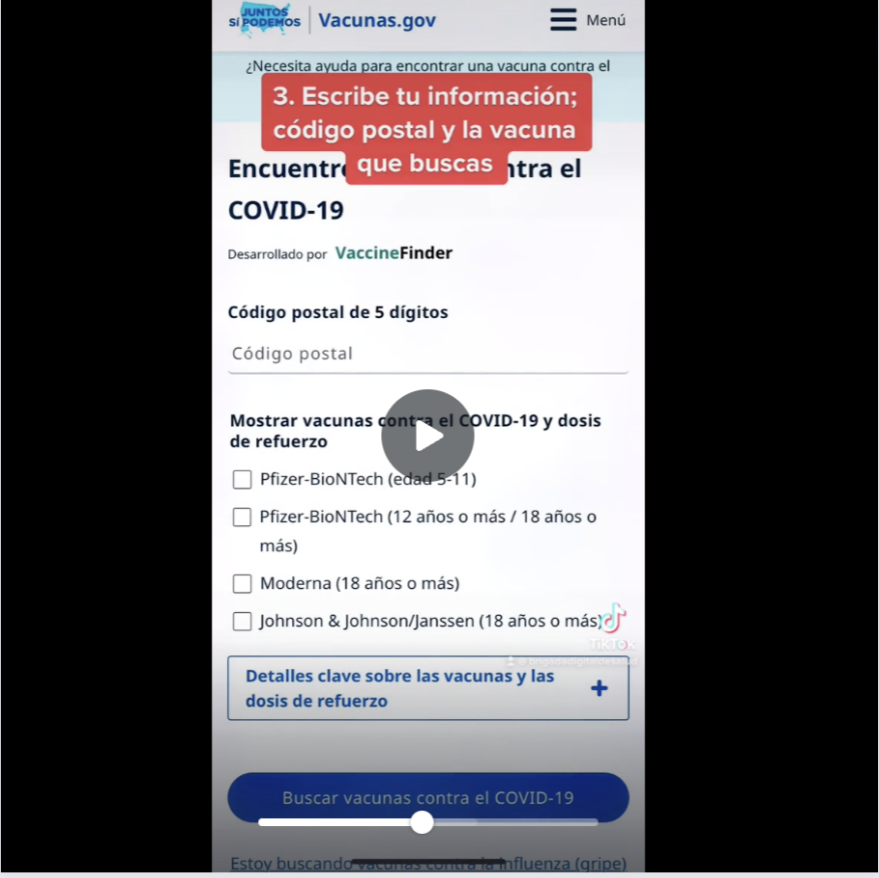
Brigada Digital TikTok video with step-by-step tutorial on navigating the Centers for Disease Control and Prevention (CDC) web-based vaccine finder in Spanish.

When promoting the use of web-based resources, posts included tools for assisting participants with navigating these resources, such as tutorials and step-by-step instructions (Figure 4).

The post content covered a wide range of topics related to COVID-19, including the science of COVID-19 and its transmission, risks, and prevention (36/101, 35.6%); COVID-19 testing (5/101, 4.9%); COVID-19 vaccine information, such as the safety and efficacy of adult and pediatric vaccines and boosters (28/101, 27.7%); risks associated with pregnancy or breastfeeding and COVID-19 (4/101, 4%); COVID-19 treatment and post–COVID-19 condition (6/101, 5.9%); and understanding COVID-19 misinformation (15/101, 14.8%). Furthermore, (7/101, 6.9%) posts were included on related topics for the purpose of participant engagement (refer to Multimedia Appendix 1 for the complete 5-week schedule of intervention post topics). The posts were scheduled to be delivered every morning, afternoon, and evening. Furthermore, the members of the group had unrestricted access to the Facebook group content, allowing them to view the posts and engage with all the content at any time.

The Facebook intervention was facilitated by 2 Spanish-speaking moderators. The moderators engaged group members in discussions about the posts by posing questions, eliciting opinions, and encouraging the sharing of relevant experiences. Group activity was monitored daily to promptly respond to comments and acknowledge group member participation in a timely manner to encourage continued engagement. Furthermore, the moderators aimed to be helpful in responding to questions by providing detailed responses and links to additional information and resources. In addition, 5 weekly polls were administered to inquire about the group members’ risk perceptions, attitudes, and vaccine intentions. Given the timing of the intervention leading up to the start of the school year for children, the poll questions inquired about parents’ concerns about sending their children back to school with COVID-19 still circulating as well as barriers and intentions to vaccinating their children. During their interactions with participants, the moderators prioritized adherence to culturally appropriate expectations of respect, attentiveness, and kindness [47-49].

### Data Collection

#### Survey

A brief, web-based survey was self-administered in Spanish using the Qualtrics software. Following approval by the George Washington University Institutional Review Board committee and participants’ informed consent, the survey was administered at 2 time points, namely at baseline and after 5 weeks. The participants were incentivized for survey completion. This paper reports intervention group participants’ survey responses at the 5-week follow-up and participants’ sociodemographics reported at baseline, including age, sex, education, income, employment status, household composition, and whether they were born in the United States.

The survey instrument included an item for self-reported adult COVID-19 vaccination, with the response options of “I received 1 dose of a 2-dose series,” “I received both doses of a 2-dose series,” “I received a one-dose vaccine (for adults only),” and “I have not been vaccinated against COVID-19.” Furthermore, a survey item for child COVID-19 vaccination was included for parents with children in the age groups of <5 years, 5 to 11 years, and 12 to 17 years, with response options similar to those provided for the item for adult COVID-19 vaccination. In addition, participants were asked whether they had been required to vaccinate.

To assess participants’ intervention exposure, 2 categorical survey items focusing on self-reported exposure asked how frequently participants visited the Brigada Digital Facebook group and saw the group posts in an average week during the intervention. The survey included standardized and open-ended items related to participants’ reactions to the intervention content, moderators, and group environment. A total of 4 categorical survey items assessed participants’ opinions regarding the information received in the group (ie, how informative or trustworthy the information was, the most useful topics, and their preferences regarding post formats); 2 categorical survey items assessed participants’ opinions about the group moderators; and 2 categorical survey items assessed participants’ opinions of the Facebook group environment and members. These items asked the participants to select their level of agreement with a series of statements, using a 5-point Likert scale ranging from *disagree* to *strongly agree*. For participant engagement, we used 3 categorical survey items focusing on self-reported engagement, asking whether they introduced themselves to the group and how frequently they reacted to and commented on posts. Furthermore, 3 open-ended items were included to inquire about participants’ reasons for not engaging in each of these actions. Finally, to elicit participants’ feedback about the intervention, we included 3 open-ended survey items that asked what they liked or did not like about the Facebook group and what suggestions they had for improving the intervention.

#### Facebook Analytics

Facebook analytics facilitated the tracking of views, reactions, and comments for each post and each participant. We assessed participants’ exposure to the intervention using the metrics of total post views and total views for each participant. We assessed participants’ engagement using the metrics of total post reactions and comments and total reactions and comments for each participant.

### Ethical Considerations

All study protocols were reviewed and approved by the George Washington University Institutional Review Board (study #NCR213586). All study participants provided informed consent before enrollment, including their acknowledgment that their survey responses were confidential and that data would be deidentified for analysis and reported in aggregate. The participants were compensated with US $65 in retail gift cards for enrolling in the group and completing the surveys.

### Data Analysis

We performed a mixed methods analysis. The survey items were assessed cross-sectionally among intervention participants (N=55) only at follow-up. The survey results for participants’ intervention exposure and reactions as well as participants’ self-reported engagement and opinions about the intervention content, moderator, and group environment were assessed using response frequencies for 13 categorical survey items. The participants’ qualitative responses to 6 open-ended survey items were thematically analyzed to identify the predominant reasons for not engaging with the intervention and areas of concordance for what was liked or disliked about the intervention and to summarize suggestions for improving the intervention. The participants’ quotes were selected and translated into English to illustrate these themes and topics.

Facebook metrics were cumulatively assessed for the duration of the intervention. The Facebook metrics of total post views, reactions, and comments were summed to further assess participants’ intervention exposure and engagement. Views, reactions, and comments were also summed for each participant, and participant averages were tabulated for each of the 3 metrics. The Stata (version 17; StataCorp) software was used for quantitative analysis, and the NVivo (version 14; QSR International) software was used for qualitative analysis.

## Results

### Overview

The 55 intervention group participants had an average age of 38 (SD 7) years, 91% (n=50) were female, 67% (n=37) had less than a college education, and 91% (n=50) were foreign born. Among the intervention group participants, 64% (n=35) reported having children aged 0 to 4 years living in their household, 64% (n=35) had children aged 5 to 11 years, and 49% (n=27) had adolescents aged 12 to 17 years. Regarding employment status and income, 44% (n=24) of the participants were employed, 49% (n=27) reported being unemployed or staying at home, and 58% (n=32) reported earning an annual household income of ≤US $35,000. At baseline, 85% (n=47) of the parent participants had received an initial 1-dose COVID-19 vaccine or completed a 2-dose vaccine series, with 29% (n=16) of the participants saying that they had been required to vaccinate. Among the intervention participants, 3% (1/35) of the parents with children aged 0 to 4 years said that their children had completed the 2-dose vaccine series, with 31% (11/35) of the parents with children aged 5 to 11 years and 70% (19/27) of the parents with adolescents aged 12 to 17 years also saying that their children had completed the 2-dose vaccine series (Table 1).

**Table 1:** Intervention group participants’ sociodemographics and COVID-19 vaccination status (N=55).

Variables		Values
**Sociodemographics**		
	Age (y), mean (SD)	38 (7)
	Sex (female), n (%)	50 (91)
	Education lesser than a bachelor’s degree, n (%)	37 (67)
	Foreign born, n (%)	50 (91)
	Employed, n (%)	24 (44)
	Unemployed or staying at home, n (%)	27 (49)
	Annual household income ≤US $35,000, n (%)	32 (58)
**COVID-19 primary vaccination series completion, n (%)**		
	Parents	47 (85)
	Children aged 0-4 y (n=35)	1 (3)
	Children aged 5-11 y (n=35)	11 (31)
	Adolescents aged 12-17 y (n=27)	19 (70)

### Participants’ Intervention Exposure

When asked how many times they visited the private Facebook group per week during the study, 40% (22/55) of the participants reported visiting the group 1 to 3 times per week, a substantial proportion (18/55, 33%) of the participants reported visiting the group 4 to 6 times per week, a smaller proportion (8/55, 15%) of the participants reported visiting <1 time per week, and an even smaller proportion (7/55, 13%) of the participants reported visiting the group ≥7 times per week (Table 2).

**Table 2:** Participants’ average weekly exposure to the intervention content (N=55).

Variables			Values, n (%)
**Number of group visits/wk**			
	1-3	22 (40)	
	4-6	18 (33)	
	<1	8 (15)	
	>7	7 (13)	
**Number of posts viewed/d**			
	1-2	23 (42)	
	3-4	16 (29)	
	<1	12 (22)	
	≥5	4 (7)	

In addition, when asked how many posts, on average, they viewed per day, 42% (23/55) of the participants reported viewing 1 to 2 posts per day, approximately one-third (16/55, 29%) of the participants reported viewing 3 to 4 posts per day, approximately one-fifth (12/55, 22%) of the participants reported viewing <1 post per day, and a small proportion (4/55, 7%) of the participants reported viewing ≥5 posts per day. Facebook group analytics from the 5-week period validated this level of participant exposure, with a total of 2004 post views or an average of 36.4 post views per participant.

### Reactions to the Intervention Content

The participants generally had positive reactions to the content of the intervention. Most participants (49/55, 89%) indicated that they agreed or *strongly agreed* that the intervention content was informative, whereas only 7% (4/55) *disagreed* or *strongly disagreed*. When asked what they liked about the intervention, 78% (43/55) of the participants responded that they liked the information provided and found it to be important and beneficial to them:

They give a lot of very important information, and more for parents, it’s super important to keep us informed.

Very good information for our benefit.

Everything. I like it because they inform us well about the vaccine.

Specifically, 13% (7/55) of the participants responded that they liked how the group content kept them informed of the latest, up-to-date information about COVID-19:

It is a very helpful group informing and trying to keep people up to date on COVID.

Furthermore, 13% (7/55) of the participants expressed that they liked that the topics were covered in detail yet in a way that was easy to comprehend, concise, and interesting. One of the participants commented on how the information provided in the group helped her make COVID-19 vaccination decisions:

All the information was explained well and the posts were understood. I had no problems understanding the topics.

I like the way the topics are fully explained.

All the information that was given to me in the group seemed very interesting to me.

This helped me be confident in decisions about the COVID vaccine.

In addition, most participants (49/55, 89%) agreed or strongly agreed that they trusted the information presented in the posts, whereas very few (2/55, 4%) *disagreed* or *strongly disagreed*. When asked what they liked about the intervention, 3 (5%) of the 55 participants echoed this viewpoint, indicating that they liked receiving “official” information about COVID-19 vaccines that was from a trusted source or supported by data:

That it addresses issues about vaccines and gives official information that many times one does not know.

That I can get information and opinions from a safe place.

I liked that they talked about the importance of vaccines with data since there are a lot of incredulous people that give out different kinds of information.

When asked for feedback on the quantity of posts received, 89% (49/55) of the participants thought that they had received the correct quantity of posts, and 11% (6/55) thought that they had not received enough posts. Furthermore, the intervention participants were asked which post topics they deemed to be the most useful. According to the participants, the 2 most useful topics were the safety and efficacy of adult COVID-19 vaccines and understanding the levels of COVID-19 risks for children, with over half (29/55, 53%) of the participants selecting each of these 2 topics (Table 3).

Furthermore, 44% (24/55) of the participants found the information about the science behind COVID-19 and its variants to be the most useful, and 38% (21/55) thought that the adult and child COVID-19 booster information was the most useful. Approximately one-fifth to one-quarter of the participants thought that the topics of COVID-19 testing (14/55, 25%), pregnancy and COVID-19 (12/55, 22%), and understanding COVID-19 misinformation (13/55, 24%) were the most useful.

When asked for feedback on how to improve the Facebook group experience, 5 (9%) of the 55 participants suggested having more content in general and continuing to provide information on COVID-19, COVID-19 vaccine side effects, COVID-19 vaccines for children, and other health topics.

Regarding preferred post types or formats, the participants indicated that they liked video content, with 51% (28/55) of the participants liking video interviews with experts, 47% (26/55) of the participants liking instructional videos, and 29% (16/55) of the participants liking audio-narrated educational posts (Table 4).

Most participants (38/55, 69%) indicated favoring educational content that could be read, and a substantial proportion also liked formats that were meant to be engaging, including group polls (25/55, 45%) and funny posts (16/55, 29%).

**Table 3:** The most useful topics covered in the posts (N=55).

Variables	Values, n (%)^a^
COVID-19 test	14 (25)
COVID-19 and pregnancy and breastfeeding	12 (22)
COVID-19 transmission and prevention	17 (31)
Information on the selection and use of masks	19 (35)
Purpose of adult and child COVID-19 boosters	21 (38)
Safety and efficacy of the COVID-19 vaccine for adults	29 (53)
Safety and efficacy of the COVID-19 vaccine for children	19 (35)
Science behind COVID-19 and its variants	24 (44)
Understanding COVID-19 risk levels for adults	20 (36)
Understanding COVID-19 risk levels for children	29 (53)
Understanding misinformation about COVID-19	13 (24)
Other topics	3 (5)

^a^The response format was *select all that apply*.

**Table 4:** Preferred post types and formats (N=55).

Variables	Values, n (%)^a^
Educational posts that could be read	38 (69)
Educational posts that were audio narrated	16 (29)
Instructional videos (eg, tutorials and demonstrations)	26 (47)
Videos of interviews or presentations with experts	28 (51)
Funny posts	16 (29)
Facebook group polls	25 (45)
“I did not like any of the posts”	2 (4)

^a^The response format was *select all that apply.*

### Reactions to the Moderators and Group Environment

When asked what they liked about the group, 7 (13%) of the 55 participants said that they liked everything about the group. Furthermore, the results revealed that most participants *agreed* or *strongly agreed* that the moderators were well informed (51/55, 93%) and helpful (50/55, 91%), whereas 2% (1/55) and 5% (3/55) *disagreed* or *strongly disagreed* with these statements, respectively. When asked what they liked about the intervention, 6 (11%) of the 55 participants specifically praised the moderators for being empathetic and responsive:

I really liked the moderator of the group.

The information that they provided and the empathy that was shown.

The information and the attentiveness to answer comments.

Regarding the group environment, a large proportion (51/55, 93%) of the participants *agreed* or *strongly agreed* that joining the group was helpful and had positive opinions about the group members. Most participants said that their perceptions of other group members were that they were friendly (45/55, 82%) and supportive (19/55, 35%) during their interactions with the group (Table 5).

**Table 5:** Participants’ opinions of other group members (N=55).

Variables	Values, n (%)^a^
Friendly	45 (82)
Bothersome	1 (2)
Supportive	19 (35)
Similar to me	18 (33)
Critical	3 (5)
Tolerant	11 (20)
Different than me	3 (5)

^a^The response format was *select all that apply.*

A little more than one-third (18/55, 33%) of the participants thought that other group members were *similar to them*; one-fifth (11/55, 20%) of the participants thought that other group members were tolerant; and few participants thought that other group members were critical (3/55, 5%), bothersome (1/55, 2%), or *different than me* (3/55, 5%).

When asked what they liked about the intervention, 2 (4%) of the 55 participants said that they liked how the group supported the Latino community with an opportunity to connect with people from many locations and with diverse ways of thinking:

I like the interest that exists to inform and support the Latino community.

It is very entertaining and friendly. You meet people from all over with different ways of thinking.

When asked whether they had recommendations for the intervention, 3 (5%) of the 55 participants indicated that they thought that the posts were not always reaching participants and suggested disseminating more posts or repeating posts:

Be more insistent in repeating the posts so that they go out to all the participants.

More posts, so if I miss one, I can look at others.

### Participants’ Engagement

The participants were asked about their average weekly engagement with the post content. The levels of engagement were relatively evenly distributed in terms of self-reported reactions to posts, with 13% (7/55) of the participants reacting to ≥5 posts in an average week, 29% (16/55) reacting to 1 to 2 posts per week, 27% (15/55) reacting to 3 to 4 posts per week, and 27% (15/55) reacting to ≤1 post per week. A small proportion (2/55, 4%) of the participants did not react to any posts. This level of participant engagement through post reactions was validated by Facebook analytics; the group had a cumulative total of 584 reactions or an average of 10.6 post reactions per participant. When asked about the reasons for having never reacted to posts, some participants indicated that they preferred to simply read the posts without reacting, whereas others said that they were simply not interested in reacting to posts.

The participants were also asked about their average weekly commenting on the posts. The levels of engagement were relatively evenly distributed in terms of self-reported commenting on posts, with 11% (6/55) of the participants commenting on posts ≥3 times in an average week, 29% (16/55) commenting on posts 1 to 2 times per week, and 36% (20/55) commenting on posts <1 time per week. Almost one-fourth (13/55, 24%) of the participants said that they never commented on posts. This level of participant engagement through post comments was validated by Facebook analytics; the group had a cumulative total of 163 comments or an average of 3 post comments per participant. When asked about the reasons for having never commented on posts, the most common reasons were that the participants preferred only receiving information from the posts, disliked commenting, or were shy.

We also inquired about self-reported group engagement, including whether participants had interacted with group members and posts. When asked whether they had introduced themselves to the group upon joining, 89% (49/55) of the participants revealed that they had. Among those who had not introduced themselves, the reasons varied. Some were interested only in staying up to date with the group’s posts and did not want to engage with others. Of the 55 participants, 2 (4%) mentioned that they were timid, whereas another 2 (4%) said they were unsure of how to introduce themselves.

When asked about recommendations for improving the intervention, 3 (5%) of the 55 participants suggested adding more opportunities for engagement, 1 (2%) requested adding more polls, and 2 (4%) suggested having opportunities to interact in real time with the moderators and group members:

Maybe they could do something to get to know each other a little more and resolve certain doubts.

## Discussion

### Principal Findings

This intervention entailed the delivery of health promotion messaging and engagement with Spanish-speaking Latino parents in a Facebook group to increase the uptake of adult and child COVID-19 vaccination. The study results suggest that this is a promising approach that should be further tested. The digital setting was particularly appealing because of the flexible opportunities it offered parents to interact both with the educational content at their own pace and with the group members and moderators to resolve concerns, ask for advice, and seek social support. This group-based approach also facilitated the use of multimedia educational formats, which included accessibility features, such as audio narration or video-based delivery, that are preferred by parents and address barriers related to literacy or health literacy.

A substantial quantity of daily posts was shared in the group over the 5-week period, with content tailored to a Latino parent audience and covering a wide range of topics related to COVID-19 risk, prevention, and vaccination. Intervention content was intended to provide updated, accurate information to influence parents’ attitudes and beliefs about adult and child COVID-19 vaccination and, ultimately, increase intentions to vaccinate. It is important to note that all content was developed before the intervention, with great attention paid to the selection and sequencing of topics, accessibility to diverse educational levels, and cultural aspects of messaging and delivery. This enabled the scheduling and delivery of many posts within a short time frame. However, given that the intervention was implemented during an ongoing pandemic, which was a rapidly evolving context, it was necessary to review and update all content immediately before scheduled delivery to ensure that the messaging was still consistent with current public health guidelines, overall risk levels, and vaccine recommendations. With the abundance of misinformation and mixed messaging on social media, this is highly important for establishing and maintaining trust and credibility in participants’ eyes [50-53]. Future interventions seeking to replicate this approach, especially in rapidly changing contexts, should also prioritize the review of scheduled content before delivery to ensure the accuracy and consistency of messaging and alignment with the latest guidelines. In addition, given that the group members resided in different areas of the United States, messaging was developed to be relevant to national COVID-19 community transmission trends and risk levels; geographically focused interventions should tailor content to reflect local circumstances more closely.

### Participants’ Intervention Exposure

The participants reported viewing posts and visiting the group a fair number of times during the intervention, with 78% (43/55) of the participants saying that they viewed at least 1 post on an average day and 85% (47/55) of the participants saying that they visited the group at least once during an average week. However, 22% (12/55) of the participants reported viewing <1 post on an average day and 15% (8/55) reported visiting the group <1 time in an average week. Although there was considerable variability in participants’ self-reported exposure to post content, posts received an average number of 36.4 views per participant during the 5 weeks.

In addition to creating content that was varied in format and tailored to the Latino parent audience, posts were scheduled to be delivered at different times of the day (10 AM, 3 PM, 5 PM, and 7 PM) to increase the likelihood that participants would receive the intervention posts in their Facebook feed, regardless of when they were using the platform. However, some participants had still not been exposed to all intervention content. This was also reflected in the participant responses, and 3 (5%) of the 55 participants stated that they thought that the posts were not always reaching group members. This could be because of a number of barriers experienced by the participants, such as competing demands for their time, or platform-specific factors, such as the Facebook algorithm for user feed content curation. Given that the Facebook algorithm is personalized and operates based on the user’s history and patterns of content interaction, more exposure will be attained based on post contents, post formats (eg, videos and photos), and posts from sources with which an individual frequently interacts [54]. For example, if intervention participants do not engage with or view posts delivered earlier in the intervention, they are less likely to be exposed to similar content in the later weeks of the intervention. Similarly, if users predominantly engage with video content, they are less likely to be exposed to content containing only images. This algorithm that dictates content in users’ feeds has implications for group-based public health interventions that rely on content exposure to improve knowledge, shift attitudes and beliefs, and increase access to resources and tools for health behavior changes. Because exposing participants to content is imperative for public health impact, future interventions could take actions to increase the likelihood that this algorithm works in their favor, such as placing content or formats that have demonstrated (or are theorized to have) higher levels of engagement at the beginning of the intervention to boost early views and engagement; sending content that varies in terms of format each day or week; and prioritizing early, active engagement strategies as well as continued engagement opportunities each week. Furthermore, as suggested by the study participants, the moderators could post content multiple times to maximize reach or use targeted engagement strategies to prompt the viewing of posts, including tagging or direct messaging specific users. In addition, the Facebook platform, in particular, has added features that allow users to customize their feed to a certain extent, including the option to select up to 30 people and pages to add to “Favorites.” Future interventions should include protocols as part of participant recruitment that request users to select the intervention group as one of their Favorites during the intervention period to increase content exposure. Furthermore, interventions can include information and prompts that explain different ways in which the group members can access content, including through their feed or direct access to the group.

### Participants’ Reactions

Overall, the Latino parent participants had positive reactions to the intervention. The participants liked various aspects of the group environment, including the opportunity to come together with other parents like them, support the Latino community, stay informed about COVID-19, and learn the perspectives and experiences of others. The participants’ perception of other group members was very positive in that they viewed others as friendly, supportive, tolerant, and “similar to them.” For future interventions that aim to recruit participants with distinctly opposing views about vaccination or any health issue with polarized viewpoints, group moderators should be trained and prepared to navigate interactions that could become less friendly and tolerant, thus potentially stifling group members’ willingness to interact. This intervention did establish group guidelines at the outset, which did not need to be reinforced at any point during the 5 weeks; however, future interventions should consider explicitly stating in the guidelines the grounds for removal from the group to prevent guideline violations.

Another aspect that appeared to contribute to participants’ positive reactions was their perceptions of the group moderators. In addition to being deemed well informed and helpful, the participants provided feedback that the moderators were attentive to the group members’ needs and questions and showed empathy. We prioritized the selection of group moderators to include individuals who were familiar with and adhered to social norms and expectations of positive interpersonal dynamics common to Latin culture, including respect for others, kindness, and attentiveness [47-49]. The moderators were also parents of children aged <18 years, enabling them to relate to the participants’ concerns about their children’s health and safety. Future interventions should seek to replicate a similar group environment with Latino parent audiences, including by having culturally competent moderators who adhere to these expectations for interactions with participants and who are highly responsive to group members’ inquiries.

Overall, participants’ feedback indicated that the intervention content was perceived to be informative and useful. There were 3 topics in particular that the participants thought were especially useful, namely the safety and efficacy of adult COVID-19 vaccines, understanding the levels of COVID-19 risks for children, and the science behind COVID-19 and its variants. Future interventions should explore these topics in depth and, as recommended by others, such as de Vere Hunt and Linos [55], should directly address COVID-19 misinformation in these areas through audience-tailored, culturally representative messaging. In addition, Latino parent participants appreciated the importance of staying up to date with COVID-19 information by regularly receiving concise content, which has also been reported elsewhere, including by Panameno and colleagues [44]. Interventions seeking to replicate this approach, especially in the context of a rapidly evolving public health emergency, should capitalize on how the interventions can address this need for parents by delivering accurate and convenient messaging at regular intervals. Furthermore, when asked what they liked about the intervention, many participants highlighted that they liked that the information was presented in detail yet in an interesting manner that was easy to comprehend. The main goal of Brigada Digital content was to address the gaps in Spanish-language COVID-19 information that was appealing to and appropriate for the audience and fully explained health and scientific concepts to build vaccine confidence. The combination of supportive, empathetic moderators with topic expertise and the delivery of content that was updated, had consistent messaging, and was accessible to audiences with diverse educational backgrounds likely contributed to 89% (49/55) of the participants saying that they trusted the information provided in the group. Source credibility and trust are paramount to effective health promotion interventions, particularly when promoting COVID-19–related behavior change [56-59]. Future interventions should work to establish this credibility and level of trust among participants by carefully considering the characteristics and selection of group moderators, fostering a welcoming group environment, and tailoring content to the specific audience.

### Participants’ Engagement

Intervention participants demonstrated an early willingness to engage with the group following recruitment, with 89% (49/55) of the participants introducing themselves to the Facebook group when prompted by the moderators. In addition, with averages of 10.6 post reactions and 3 post comments per participant, engagement was similar to or higher than that with other parent-focused Facebook interventions [56,60], although comparable interventions reaching Latino parents specifically are very limited. Combined with positive feedback regarding the group dynamics, these are promising signs of Latino parents’ willingness to engage with Brigada Digital content and learn about and discuss health-related topics in this digital group format. Similar willingness has been observed in other Latino parent–focused digital health promotion interventions [61]. The moderators of future interventions should aim to similarly establish rapport and expectations for group interactions that can promote participants’ willingness to engage with the group. This rapport might be further fostered by offering additional opportunities for engagement throughout the intervention, for example, through live discussions and events, as suggested by the participants, or through polls, which almost half of the participants said that they preferred. The kind of engagement observed among the intervention participants through platform analytics as well as from participant self-report is consistent with typical patterns of engagement among social media platform users, including Latino individuals, who are most likely to simply view content and less likely to comment on posts than react [62,63]. Future efforts might explore ways to boost more active engagement, including using polls as catalysts for discussion or addressing individuals directly with targeted engagement tactics. Moreover, based on feedback from the participants, future interventions should explore ways to offer more opportunities to interact with and get to know group moderators and members, which would further develop rapport, establish trust in the information provided, address parental concerns, and increase engagement by making experts available to answer questions that are of the most interest to participants.

In addition, delivering content in a wide variety of formats and then inquiring about preferences helped us hone in on formats that participants gravitated toward the most. Similar to the study by Panameno and colleagues [44], which demonstrated Latino parents’ preferences for COVID-19 educational information in video formats from trusted sources, approximately half (28/55, 51%) of the study participants indicated that video interviews with experts were preferred, as were instructional videos, which often portrayed a Spanish-speaking health professional giving a demonstration. Formats intended to bridge potential literacy barriers, such as videos with audio-narration features, were also well received. One surprising finding, given social media audiences’ increasing affinity for multimedia content, was that most parents liked educational posts they could read; it is possible that the inclusion of an audio-narration feature helped increase parents’ preferences for these types of posts. This may also indicate that parent audiences interested in a particular health topic, especially for their children’s well-being, may be willing to invest time and effort in reading social media content if they perceive the content to be beneficial, trustworthy, and valuable to them. Implementers of future social media group–based interventions should also aim to offer a diversity of content delivery formats, with an emphasis on video and other multimedia formats” (in consonance with “content delivery formats”) accessible to audiences with diverse levels of literacy and health literacy.

### Limitations

This study has some limitations that should be considered when interpreting the results. This study included a relatively small sample (N=55) of Spanish-speaking Latino parents, with an overrepresentation of female participants. Therefore, the results may not be generalizable to a broader population of Latino parents, particularly fathers. In addition, the study was only 5 weeks in duration, potentially limiting insights into the sustainability of participant engagement and the long-term impact on vaccine uptake. Furthermore, given that the participants were recruited through their response to Facebook advertisements, there is a potential for selection bias that could have resulted in a sample that was more interested in health-related topics and COVID-19 in particular, was more supportive of vaccination in general, and had the time and ability to participate in the group. In addition, it was not possible for this pilot study to fully track all participants’ engagement, and we did not track Facebook log-ins, the length of time using the platform, or the number of times accessing the group. Besides platform metrics of active participant exposure and engagement, such as views, reactions, and comments, data were limited to participant self-reports of group visits and post views.

### Conclusions

The findings of this study highlight the promise of a Facebook group–based intervention approach to engage Spanish-speaking Latino parents in health promotions related to COVID-19 and vaccination. The intervention participants experienced adequate exposure to the intervention content and engagement and reported overall positive reactions to the intervention. Future efforts should consider using this digital health promotion approach while integrating strategies to augment participants’ exposure to health promotion content; cultivating regular participant engagement, including using concise, audience-tailored messaging; and moderating the group with culturally aligned facilitators to foster trust, address misinformation, and promote child vaccine acceptance among parents.

## Appendix

Multimedia Appendix 1 Schedule of posts.

## Data Availability

The data sets generated and analyzed during this study are available from the corresponding author upon reasonable request.

## References

[1] de Ramos IP, Lazo M, Schnake-Mahl A, Li R, Martinez-Donate AP, Roux AV, Bilal U (2022). COVID-19 outcomes among the Hispanic population of 27 large US cities, 2020-2021. Am J Public Health.

[2] Mackey K, Ayers CK, Kondo KK, Saha S, Advani SM, Young S, Spencer H, Rusek M, Anderson J, Veazie S, Smith M, Kansagara D (2021). Racial and ethnic disparities in COVID-19-related infections, hospitalizations, and deaths: a systematic review. Ann Intern Med.

[3] Acosta AM, Garg S, Pham H, Whitaker M, Anglin O, O'Halloran A, Milucky J, Patel K, Taylor C, Wortham J, Chai SJ, Kirley PD, Alden NB, Kawasaki B, Meek J, Yousey-Hindes K, Anderson EJ, Openo KP, Weigel A, Monroe ML, Ryan P, Reeg L, Kohrman A, Lynfield R, Bye E, Torres S, Salazar-Sanchez Y, Muse A, Barney G, Bennett NM, Bushey S, Billing L, Shiltz E, Sutton M, Abdullah N, Talbot HK, Schaffner W, Ortega J, Price A, Fry AM, Hall A, Kim L, Havers FP (2021). Racial and ethnic disparities in rates of COVID-19-associated hospitalization, intensive care unit admission, and in-hospital death in the united states from march 2020 to February 2021. JAMA Netw Open.

[4] Scheiber A, Prinster TB, Stecko H, Wang T, Scott S, Shah SH, Wyne K (2023). COVID-19 vaccination rates and vaccine hesitancy among Spanish-speaking free clinic patients. J Community Health.

[5] Risk for COVID-19 infection, hospitalization, and death by race/ethnicity. Centers for Disease Control and Prevention.

[6] Do DP, Frank R (2021). Using race- and age-specific COVID-19 case data to investigate the determinants of the excess COVID-19 mortality burden among Hispanic Americans. DemRes.

[7] McFadden SM, Demeke J, Dada D, Wilton L, Wang M, Vlahov D, Nelson LE (2022). Confidence and hesitancy during the early roll-out of COVID-19 vaccines among black, Hispanic, and undocumented immigrant communities: a review. J Urban Health.

[8] Percent of people receiving COVID-19 vaccine by race/ethnicity and date administered, United States. December 14, 2020 - March 15, 2023. Center for Disease Control and Prevention.

[9] Murthy BP, Fast HE, Zell E, Murthy N, Meng L, Shaw L, Vogt T, Chatham-Stephens K, Santibanez TA, Gibbs-Scharf L, Harris LQ (2023). COVID-19 vaccination coverage and demographic characteristics of infants and children aged 6 months-4 years - United States, June 20-December 31, 2022. MMWR Morb Mortal Wkly Rep.

[10] Valier MR, Elam-Evans LD, Mu Y, Santibanez TA, Yankey D, Zhou T, Pingali C, Singleton JA (2023). Racial and ethnic differences in COVID-19 vaccination coverage among children and adolescents aged 5-17 years and parental intent to vaccinate their children - national immunization survey-child COVID module, United States, December 2020-September 2022. MMWR Morb Mortal Wkly Rep.

[11] DeVille JG, Song E, Ouellette CP COVID-19: management in children. UpToDate®.

[12] Kim L, Whitaker M, O'Halloran A, Kambhampati A, Chai SJ, Reingold A, Armistead I, Kawasaki B, Meek J, Yousey-Hindes K, Anderson EJ, Openo KP, Weigel A, Ryan P, Monroe ML, Fox K, Kim S, Lynfield R, Bye E, Shrum Davis S, Smelser C, Barney G, Spina NL, Bennett NM, Felsen CB, Billing LM, Shiltz J, Sutton M, West N, Talbot HK, Schaffner W, Risk I, Price A, Brammer L, Fry AM, Hall AJ, Langley GE, Garg S, COVID-NET Surveillance Team (2020). MMWR Morb Mortal Wkly Rep.

[13] Fleming-Dutra KE (2022). COVID-19 epidemiology in children ages 6 months– 4 years. Centers for Disease Control and Prevention Center for Immunization and Respiratory Diseases.

[14] Lee EH, Kepler KL, Geevarughese A, Paneth-Pollak R, Dorsinville MS, Ngai S, Reilly KH (2020). Race/ethnicity among children with COVID-19-associated multisystem inflammatory syndrome. JAMA Netw Open.

[15] COVID data tracker. Centers for Disease Control and Prevention.

[16] Vicetti Miguel CP, Dasgupta-Tsinikas S, Lamb GS, Olarte L, Santos RP (2022). Race, ethnicity, and health disparities in US children with COVID-19: a review of the evidence and recommendations for the future. J Pediatric Infect Dis Soc.

[17] Garcini LM, Ambriz AM, Vázquez AL, Abraham C, Sarabu V, Abraham C, Lucas-Marinelli AK, Lill S, Tsevat J (2022). Vaccination for COVID-19 among historically underserved Latino communities in the United States: perspectives of community health workers. Front Public Health.

[18] Rane MS, Kochhar S, Poehlein E, You W, Robertson MM, Zimba R, Westmoreland DA, Romo ML, Kulkarni SG, Chang M, Berry A, Parcesepe AM, Maroko AR, Grov C, Nash D, CHASING COVID Cohort Study Team (2022). Determinants and trends of COVID-19 vaccine hesitancy and vaccine uptake in a national cohort of US adults: a longitudinal study. Am J Epidemiol.

[19] Kricorian K, Turner K (2021). COVID-19 vaccine acceptance and beliefs among black and Hispanic Americans. PLoS One.

[20] Fisher C, Bragard E, Madhivanan P (2023). COVID-19 vaccine hesitancy among economically marginalized Hispanic parents of children under five years in the United States. Vaccines (Basel).

[21] Fisher CB, Bragard E, Jaber R, Gray A (2022). COVID-19 vaccine hesitancy among parents of children under five years in the United States. Vaccines (Basel).

[22] Hammershaimb EA, Cole LD, Liang Y, Hendrich MA, Das D, Petrin R, Cataldi JR, O'Leary ST, Campbell JD (2022). COVID-19 vaccine acceptance among US parents: a nationally representative survey. J Pediatric Infect Dis Soc.

[23] Khubchandani J, Macias Y (2021). COVID-19 vaccination hesitancy in Hispanics and African-Americans: a review and recommendations for practice. Brain Behav Immun Health.

[24] Bleakley A, Hennessy M, Maloney E, Young DG, Crowley J, Silk K, Langbaum JB (2022). Psychosocial determinants of COVID-19 vaccination intention among white, black, and Hispanic adults in the US. Ann Behav Med.

[25] Silesky MD, Panchal D, Fields M, Peña AS, Diez M, Magdaleno A, Frausto-Rodriguez P, Bonnevie E (2023). A multifaceted campaign to combat COVID-19 misinformation in the Hispanic community. J Community Health.

[26] Aleksandric A, Anderson HI, Melcher S, Nilizadeh S, Wilson GM (2022). Spanish Facebook posts as an indicator of COVID-19 vaccine hesitancy in Texas. Vaccines (Basel).

[27] Druckman J, Ognyanova K, Baum M, Lazer D, Perlis R, Volpe JD, Santillana M, Chwe H, Quintana A, Simonson M The role of race, religion, and partisanship in misinformation about COVID-19. Northwestern Institute for Policy Research.

[28] Fisher CB, Gray A, Sheck I (2021). COVID-19 pediatric vaccine hesitancy among racially diverse parents in the United States. Vaccines (Basel).

[29] Lopes L, Hamel L, Sparks G, Montero A, Presiado M, Brody M KFF COVID-19 vaccine monitor: July 2022. Kaiser Family Foundation.

[30] Baumer-Mouradian SH, Hart RJ, Visotcky A, Fraser R, Prasad S, Levas M, Nimmer M, Brousseau DC (2022). Understanding influenza and SARS-CoV-2 vaccine hesitancy in racial and ethnic minority caregivers. Vaccines (Basel).

[31] Scott VP, Hiller-Venegas S, Edra K, Prickitt J, Esquivel Y, Melendrez B, Rhee KE (2022). Factors associated with COVID-19 vaccine intent among Latino SNAP participants in Southern California. BMC Public Health.

[32] Cascini F, Pantovic A, Al-Ajlouni YA, Failla G, Puleo V, Melnyk A, Lontano A, Ricciardi W (2022). Social media and attitudes towards a COVID-19 vaccination: a systematic review of the literature. EClinicalMedicine.

[33] Wilson SL, Wiysonge C (2020). Social media and vaccine hesitancy. BMJ Glob Health.

[34] Pierri F, Perry BL, DeVerna MR, Yang K, Flammini A, Menczer F, Bryden J (2022). Online misinformation is linked to early COVID-19 vaccination hesitancy and refusal. Sci Rep.

[35] Clark SE, Bledsoe MC, Harrison CJ (2022). The role of social media in promoting vaccine hesitancy. Curr Opin Pediatr.

[36] Jennings W, Stoker G, Bunting H, Valgarðsson VO, Gaskell J, Devine D, McKay L, Mills MC (2021). Lack of trust, conspiracy beliefs, and social media use predict COVID-19 vaccine hesitancy. Vaccines (Basel).

[37] Loomba S, de Figueiredo A, Piatek SJ, de Graaf K, Larson HJ (2021). Measuring the impact of COVID-19 vaccine misinformation on vaccination intent in the UK and USA. Nat Hum Behav.

[38] Roozenbeek J, Schneider CR, Dryhurst S, Kerr J, Freeman AL, Recchia G, van der Bles AM, van der Linden S (2020). Susceptibility to misinformation about COVID-19 around the world. R Soc Open Sci.

[39] Romer D, Winneg KM, Jamieson PE, Brensinger C, Jamieson KH (2022). Misinformation about vaccine safety and uptake of COVID-19 vaccines among adults and 5-11-year-olds in the United States. Vaccine.

[40] Herrera-Peco I, Jiménez-Gómez B, Peña Deudero JJ, Benitez De Gracia E, Ruiz-Núñez C (2021). Healthcare professionals' role in social media public health campaigns: analysis of Spanish pro vaccination campaign on Twitter. Healthcare (Basel).

[41] Demeke J, Ramos SR, McFadden SM, Dada D, Nguemo Djiometio J, Vlahov D, Wilton L, Wang M, Nelson LE (2023). Strategies that promote equity in COVID-19 vaccine uptake for Latinx communities: a review. J Racial Ethn Health Disparities.

[42] Abdul-Mutakabbir JC, Granillo C, Peteet B, Dubov A, Montgomery SB, Hutchinson J, Casey S, Simmons K, Fajardo A, Belliard JC (2022). Rapid implementation of a community-academic partnership model to promote COVID-19 vaccine equity within racially and ethnically minoritized communities. Vaccines (Basel).

[43] Ramirez AG, Despres C, Chalela P, Weis J, Sukumaran P, Munoz E, McAlister AL (2022). Pilot study of peer modeling with psychological inoculation to promote coronavirus vaccination. Health Educ Res.

[44] Panameno M, Blanco LR, Hernandez AM, Escobar R, Zendejas B, Rafaela S, Castellon-Lopez YM (2023). Using digital technology to build COVID-19 vaccine confidence: a qualitative study among Latinx parents of children aged 5-11 in under-resourced communities across Los Angeles county. Vaccines (Basel).

[45] Andrade EL, Abroms LC, González AI, Favetto C, Gomez V, Díaz-Ramírez M, Palacios C, Edberg MC (2023). Assessing Brigada Digital de Salud audience reach and engagement: a digital community health worker model to address COVID-19 misinformation in Spanish on social media. Vaccines (Basel).

[46] Ajzen I (1991). The theory of planned behavior. Organ Behav Hum Decis Process.

[47] Magaña D (2020). Local voices on health care communication issues and insights on Latino cultural constructs. Hisp J Behav Sci.

[48] Calzada EJ, Fernandez Y, Cortes DE (2010). Incorporating the cultural value of respeto into a framework of Latino parenting. Cultur Divers Ethnic Minor Psychol.

[49] García AA, Zuñiga JA, Lagon C (2017). A personal touch: the most important strategy for recruiting Latino research participants. J Transcult Nurs.

[50] Seeger MW (2006). Best practices in crisis communication: an expert panel process. J Appl Commun Res.

[51] Savoia E, Lin L, Viswanath K (2013). Communications in public health emergency preparedness: a systematic review of the literature. Biosecur Bioterror.

[52] Covello VT (2003). Best practices in public health risk and crisis communication. J Health Commun.

[53] (2014). Crisis and emergency risk communication manual. Centers for Disease Control and Prevention.

[54] Why are posts I’ve already seen still appearing in my Facebook feed?. Facebook.

[55] de Vere Hunt I, Linos E (2022). Social media for public health: framework for social media-based public health campaigns. J Med Internet Res.

[56] Buller D, Walkosz B, Henry K, Woodall WG, Pagoto S, Berteletti J, Kinsey A, Divito J, Baker K, Hillhouse J (2022). Promoting social distancing and COVID-19 vaccine intentions to mothers: randomized comparison of information sources in social media messages. JMIR Infodemiology.

[57] Barua Z (2022). COVID-19 misinformation on social media and public’s health behavior: understanding the moderating role of situational motivation and credibility evaluations. Hu Arenas (Forthcoming).

[58] Shah Z, Wei L (2022). Source credibility and the information quality matter in public engagement on social networking sites during the COVID-19 crisis. Front Psychol.

[59] Conlin J, Baker M, Zhang B, Shoenberger H, Shen F (2023). Facing the strain: the persuasive effects of conversion messages on COVID-19 vaccination attitudes and behavioral intentions. Health Commun.

[60] Epstein M, Oesterle S, Haggerty KP (2019). Effectiveness of Facebook groups to boost participation in a parenting intervention. Prev Sci.

[61] Rojas LM, Bahamon M, Lebron C, Montero-Zamora P, Pardo M, Wakefield M, Tapia M, Estrada Y, Schwartz SJ, Pantin H (2021). A feasibility trial of an online-only, family-centered preventive intervention for Hispanics: e-familias unidas. J Prim Prev.

[62] Rivera YM, Moran MB, Thrul J, Joshu C, Smith KC (2022). Contextualizing engagement with health information on Facebook: using the social media content and context elicitation method. J Med Internet Res.

[63] Goldsmith LP, Rowland-Pomp M, Hanson K, Deal A, Crawshaw AF, Hayward SE, Knights F, Carter J, Ahmad A, Razai M, Vandrevala T, Hargreaves S (2022). Use of social media platforms by migrant and ethnic minority populations during the COVID-19 pandemic: a systematic review. BMJ Open.

