# Correction: Latino Parents’ Reactions to and Engagement With a Facebook Group–Based COVID-19 Vaccine Promotion Intervention: Mixed Methods Pilot Study

**DOI:** 10.2196/76107

**Published:** 2025-04-28

**Authors:** Anna I González-Salinas, Elizabeth L Andrade, Lorien C Abroms, Kaitlyn Gómez, Carla Favetto, Valeria M Gómez, Karen K Collins

**Affiliations:** 1 George Washington University Washington, DC United States; 2 California State University Fullerton, CA United States

In “Latino Parents’ Reactions to and Engagement With a Facebook Group–Based COVID-19 Vaccine Promotion Intervention: Mixed Methods Pilot Study” (JMIR Form Res 2024;8:e51331) the authors made one update.

The Acknowledgements section has been changed from:

This research was funded by the National Institutes of Health Community Engagement Alliance Against COVID-19 Disparities (NIH CEAL; grant 1OT2HL156812-01; multiple Principal Investigators: Mark Edberg and ELA), and the George Washington University Institute for Data, Democracy, and Politics (internal grant; multiple Principal Investigators: LCA and ELA).

To:

This research was, in part, funded by the National Institutes of Health (NIH) Agreement OT2HL158287. The views and conclusions contained in this document are those of the authors and should not be interpreted as representing the official policies, either expressed or implied, of the NIH. This research was also funded, in part, by The George Washington University Institute for Data, Democracy, and Politics (multiple Principal Investigators LCA and ELA).

The correction will appear in the online version of the paper on the JMIR Publications website, together with the publication of this correction notice. Because this was made after submission to PubMed, PubMed Central, and other full-text repositories, the corrected article has also been resubmitted to those repositories.

